# Coenzyme Q_10_ Biosynthesis Established in the Non-Ubiquinone Containing *Corynebacterium glutamicum* by Metabolic Engineering

**DOI:** 10.3389/fbioe.2021.650961

**Published:** 2021-03-30

**Authors:** Arthur Burgardt, Ayham Moustafa, Marcus Persicke, Jens Sproß, Thomas Patschkowski, Joe Max Risse, Petra Peters-Wendisch, Jin-Ho Lee, Volker F. Wendisch

**Affiliations:** ^1^Genetics of Prokaryotes, Faculty of Biology and Center for Biotechnology (CeBiTec), Bielefeld University, Bielefeld, Germany; ^2^Technology Platform Genomics, Center for Biotechnology (CeBiTec), Bielefeld University, Bielefeld, Germany; ^3^Industrial Organic Chemistry and Biotechnology, Department of Chemistry, Bielefeld University, Bielefeld, Germany; ^4^Fermentation Technology, Technical Faculty and Center for Biotechnology (CeBiTec), Bielefeld University, Bielefeld, Germany; ^5^Major in Food Science & Biotechnology, School of Food Biotechnology & Nutrition, Kyungsung University, Busan, South Korea

**Keywords:** coenzyme Q_10_ (CoQ 10), *Corynebacterium glutamicum*, metabolic engineering, isoprenoids, aromatic compounds, fermentation

## Abstract

Coenzyme Q_10_ (CoQ10) serves as an electron carrier in aerobic respiration and has become an interesting target for biotechnological production due to its antioxidative effect and benefits in supplementation to patients with various diseases. For the microbial production, so far only bacteria have been used that naturally synthesize CoQ10 or a related CoQ species. Since the whole pathway involves many enzymatic steps and has not been fully elucidated yet, the set of genes required for transfer of CoQ10 synthesis to a bacterium not naturally synthesizing CoQ species remained unknown. Here, we established CoQ10 biosynthesis in the non-ubiquinone-containing Gram-positive *Corynebacterium glutamicum* by metabolic engineering. CoQ10 biosynthesis involves prenylation and, thus, requires farnesyl diphosphate as precursor. A carotenoid-deficient strain was engineered to synthesize an increased supply of the precursor molecule farnesyl diphosphate. Increased farnesyl diphosphate supply was demonstrated indirectly by increased conversion to amorpha-4,11-diene. To provide the first CoQ10 precursor decaprenyl diphosphate (DPP) from farnesyl diphosphate, DPP synthase gene *ddsA* from *Paracoccus denitrificans* was expressed. Improved supply of the second CoQ10 precursor, *para*-hydroxybenzoate (pHBA), resulted from metabolic engineering of the shikimate pathway. Prenylation of pHBA with DPP and subsequent decarboxylation, hydroxylation, and methylation reactions to yield CoQ10 was achieved by expression of *ubi* genes from *Escherichia coli*. CoQ10 biosynthesis was demonstrated in shake-flask cultivation and verified by liquid chromatography mass spectrometry analysis. To the best of our knowledge, this is the first report of CoQ10 production in a non-ubiquinone-containing bacterium.

## Introduction

Coenzyme Q_10_ (CoQ10), also referred to as ubiquinone-10, is a lipid-soluble quinone (CoQ) that serves as an electron carrier in the electron transport chain of aerobic respiration and is widely distributed among organisms (Kawamukai, 2002). Besides its function in cell respiration, it is known to act as an antioxidant by protection of lipids against peroxidation and prevention of oxidative damage to mitochondrial proteins and DNA (Ernster and Dallner, 1995), a property that makes it interesting as an agent against skin aging in the cosmetic industry (Vinson and Anamandla, 2006). Medical studies showed that the supplementation of CoQ10 may be beneficial for patients with diabetes and heart failures (Hodgson et al., 2002; Weant and Smith, 2005) as well as for patients with neurologic diseases like Alzheimer’s and Parkinson’s disease (Yang et al., 2010; Mischley et al., 2012).

Coenzyme Q_10_ consists of the aromatic 2,3-dimethoxy-5-methyl-benzoquinone and a side chain of ten isoprenoid units. It is naturally synthesized from the two precursors decaprenyl diphosphate (DPP) and *para*-hydroxybenzoate (pHBA). The length of the polyprenyl diphosphate determines the CoQ species in *E. coli* (Asai et al., 1994). The condensation of octaprenyl diphosphate at the C3 position of pHBA is mediated by octaprenyltransferase UbiA and several subsequent modifications at the aromatic ring yield coenzyme Q_8_ (CoQ8) (Young et al., 1972). In fact, UbiA promiscuously recognizes isoprenoid diphosphates of different lengths (Suzuki et al., 1994; Cheng and Li, 2014), which enabled production of CoQ10 in *E. coli* by merely expressing *ddsA* from *P. denitrificans*, coding for decaprenyl diphosphate synthase, to provide the precursor DPP (Takahashi et al., 2003).

**FIGURE 1:**
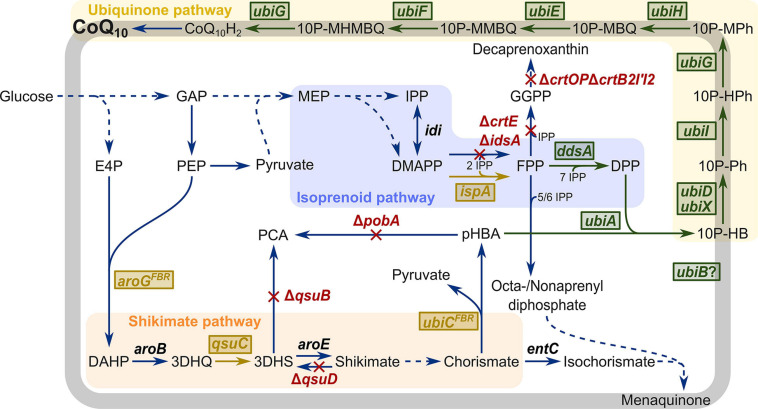
Schematic overview of metabolic engineering of *C. glutamicum* for CoQ10 production. Heterologous plasmid-driven expression of genes is visualized in green boxes. Chromosomal overexpression of native and heterologous genes is visualized in yellow boxes. Deletion of genes is visualized in red. E4P, erythrose 4-phosphate; DAHP, 3-deoxy-D-arabinoheptulosonate 7-phosphate; 3DHQ, 3-dehydroquinate; 3DHS, 3-dehydroshikimic acid; PCA, protocatechuic acid; pHBA, *para*-hydroxybenzoate; GAP, glyceraldehyde 3-phosphate; PEP, phosphoenolpyruvate; MEP, 2-C-methylerythritol 4-phosphate; IPP, isopentenyl diphosphate; DMAPP, dimethylallyl diphosphate; GPP, geranyl diphosphate; FPP, farnesyl diphosphate; GGPP, geranylgeranyl diphosphate; DPP, decaprenyl diphosphate, 10P-HB, 3-decaprenyl-4-hydroxybenzoate; 10P-Ph, 2-decaprenylphenol; 10P-HPh, 2-decaprenyl-6-hydroxyphenol; 10P-MPh, 2-decaprenyl-6-methoxyphenol; 10P-MBQ, 2-decaprenyl-6-methoxy-1,4-benzoquinol; 10P-MMBQ, 2-decaprenyl-3-methyl-6-methoxy-1,4-benzoquinol; 10P-MHMBQ, 2-decaprenyl-3-methyl-5-hydroxy-6-methoxy-1,4-benzoquinol; CoQ_10_H_2_, ubiquinol-10; CoQ_10_, ubiquinone-10; *aroG*^*FBR*^, feedback-resistant DAHP synthase from *E. coli*; *aroB*, 3-dehydroquinate synthase; *qsuC*, 3-dehydroquinate dehydratase; *qsuB*, 3-dehydroshikimate dehydratase; *qsuD*, shikimate dehydrogenase; *aroE*, shikimate dehydrogenase; *ubiC*^*FBR*^, feedback-resistant chorismate-pyruvate lyase from *E. coli*; *entC*, isochorismate synthase; *pobA, para*-hydroxybenzoate hydroxylase; *idi*, isopentenyl diphosphate isomerase; *crtE*, geranylgeranyl diphosphate synthase; *idsA*, geranylgeranyl diphosphate synthase; *ispA*, farnesyl diphosphate synthase; *crtOP*, cg0717-cg0723; *crtB2I’I2*, cg2668-cg2672; *ddsA*, decaprenyl diphosphate synthase from *P. denitrificans*; *ubiA*, 4-hydroxybenzoate octaprenyltransferase; *ubiD*, 3-octaprenyl-4-hydroxybenzoate decarboxylase; *ubiX*, flavin prenyltransferase; *ubiI*, 2-octaprenylphenol hydroxylase; *ubiG*, 2-octaprenyl-6-hydroxyphenol/2-octaprenyl-3-methyl-5-hydroxy-6-methoxy-1,4-benzoquinol methyltransferase; *ubiH*, 2-octaprenyl-6-methoxyphenol hydroxylase; *ubiE*, ubiquinone/menaquinone biosynthesis methyltransferase; *ubiF*, 2-octaprenyl-3-methyl-6-methoxy-1,4-benzoquinol hydroxylase; *ubiB*, probably protein kinase, function unknown.

The precursors for CoQ10, DPP, and pHBA are derived from the isoprenoid and shikimate pathways, respectively (Figure 1). The isoprenoid diphosphate DPP is synthesized in the 2-*C*-methyl-D-erythritol 4-phosphate (MEP) pathway that yields the two isomers dimethylallyl diphosphate (DMAPP) and isopentenyl diphosphate (IPP). In archaea, bacteria, and eukarya, they serve as the basis for a variety of isoprenoids like sterols, carotenoids, and ubiquinone, menaquinone, or secondary metabolites like mono-, sesqui-, and diterpenes (Rohmer and Rohmer, 1999). The *E. coli* farnesyl diphosphate (FPP) synthase IspA catalyzes the condensation of two IPP units with DMAPP or one IPP unit with geranyl diphosphate (GPP) to FPP (Fujisaki et al., 1990). Polyprenyltransferases add IPP units to FPP to generate polyprenyl diphosphates of various lengths. In CoQ10-containing bacteria such as *P. denitrificans, Agrobacterium tumefaciens*, and *Rhodobacter sphaeroides*, DPP is synthesized from FPP, e.g., by decaprenyl diphosphate synthase DdsA (Collins and Jones, 1981). The aromatic pHBA is synthesized from the shikimate pathway metabolite chorismate by chorismate-pyruvate lyase UbiC. The UbiC enzyme from *E. coli* shows product inhibition; however, this could be overcome in the feedback-resistant mutant UbiC^*L31A*^. Use of UbiC^*L31A*^ increased production of pHBA by engineered *C. glutamicum* by about 55% as compared to wild-type UbiC (Purwanto et al., 2018).

As a key step in CoQ10 synthesis, pHBA is prenylated with DPP by a 4-hydroxybenzoate polyprenyltransferase, yielding 3-decaprenyl-4-hydroxybenzoate (Takahashi et al., 2003). This intermediate is then modified in a series of reactions involving a decarboxylation, three hydroxylation, and three methylation steps (Figure 1 and Supplementary Figure 1). In *E. coli*, enzymes UbiD, UbiI, UbiG, UbiH, UbiE, and UbiF are known to catalyze these reactions; however, the functions of other proteins such as UbiB, UbiJ, and UbiK in CoQ8 biosynthesis have not yet been fully elucidated (Aussel et al., 2014b). Because *ubiX* and *ubiD* mutants both accumulated 3-octaprenyl-4-hydroxybenzoate and produced low levels of CoQ8, both gene products were thought to act as decarboxylases (Gulmezian et al., 2007), More recently, it was discovered that UbiX actually prenylates flavin mononucleotide with DMAP to generate a cofactor required for UbiD activity (White et al., 2015). The first hydroxylation step is catalyzed by UbiI (not by UbiB as formerly believed) (Hajj Chehade et al., 2013). UbiB lacks conserved motifs characteristic for hydroxylases but might act as a putative kinase that is involved in CoQ synthesis; however, studies on UbiB activity are lacking (Hajj Chehade et al., 2013). To date, the catalytic mechanism of UbiJ and UbiK is not fully understood, but mutants of *ubiJ* and *ubiK* showed decreased amounts of CoQ8 and accumulation of the precursor octaprenylphenol. Additionally, UbiJ and UbiK form a complex that interacts with the major lipid palmitoleic acid in *E. coli* (Loiseau et al., 2017). While the catalytic functions of many of these enzymes are known, the roles of UbiB, UbiJ, and UbiK remain elusive. Thus, since the set of enzymes required and sufficient for CoQ synthesis is not known, approaches to biotechnological CoQ10 production were hitherto based on microorganisms that natively contain ubiquinones and, thus, possess all necessary genes for CoQ biosynthesis.

Biotechnological CoQ10 production was achieved after classical mutagenesis and screening of native CoQ10-producing bacteria like *A. tumefaciens* and *R. sphaeroides*, (Ha et al., 2008; Kien et al., 2010; Yuan et al., 2012). These bacteria were also subjected to metabolic engineering (Koo et al., 2010; Lu et al., 2015; Zhang et al., 2018; Chen et al., 2019) as well as the model bacterium *E. coli* (Takahashi et al., 2003; Park et al., 2005; Zahiri et al., 2006). Metabolically engineered *A. tumefaciens* and *R. sphaeroides* strains outperformed engineered *E. coli* strains with regard to CoQ10 production. For instance, cellular CoQ10 contents of 4.59 mg g^–1^ DCW (Zhang et al., 2018) and 12.94 mg g^–1^ DCW (Lu et al., 2015) were achieved with metabolically engineered *R. spharoides* strains as compared to 0.29 mg g^–1^ DCW (Park et al., 2005) and 2.43 mg g^–1^ DCW (Zahiri et al., 2006) with recombinant *E. coli* strains.

Electron carriers in the electron transport chain of aerobic bacteria that lack CoQ are, for example, menaquinones (MK) and dihydromenaquinones [MK(H2)] (Collins et al., 1977; Collins and Jones, 1981). The Gram-positive, rod-shaped bacterium *C. glutamicum* belongs to this type of bacteria, and it possesses MK9(H2) and MK8(H2) as electron carriers. Similar to ubiquinones, dihydromenaquinones contain an aromatic and a prenyl moiety. The aromatic moiety in MK is a bicyclic menaquinone, not a monocyclic ubiquinone. Moreover, as opposed to CoQ biosynthesis, the aromatic precursor is almost fully modified before being prenylated in the second to last step of MK biosynthesis (Meganathan and Kwon, 2009). *C. glutamicum* is used in the food and feed industry for the million-ton-scale amino acid production (Eggeling and Bott, 2015; Wendisch, 2020). *C. glutamicum* shows stable growth to high cell densities (Riesenberg and Guthke, 1999; Pfeifer et al., 2017) and has been engineered for production of, e.g., *N*-functionalized amino acids (Mindt et al., 2020), diamines (Wendisch et al., 2018; Chae et al., 2020), alcohols (Jojima et al., 2015; Siebert and Wendisch, 2015), and organic acids (Wieschalka et al., 2013; Purwanto et al., 2018). With respect to production of molecules originating from the shikimate or isoprenoid pathways, metabolic engineering enabled production of terpenoids such as pinene (Kang et al., 2014), patchoulol (Henke et al., 2018), valencene (Frohwitter et al., 2014), or various carotenoids (Heider et al., 2014b,c; Henke et al., 2016) and of aromatic or aromatic-derived compounds such as muconic acid (Becker et al., 2018; Shin et al., 2018), phenylpropanoids (Kallscheuer et al., 2016, 2017), 4-aminobenzoate (Kubota et al., 2016), shikimate (Sato et al., 2020), protocatechuate (Kogure et al., 2020), 4-hydroxybenzyl alcohol (Kim et al., 2020), and pHBA (Kitade et al., 2018; Purwanto et al., 2018).

Therefore, in this work, in a step-by-step approach CoQ10 biosynthesis was enabled in *C. glutamicum*. Based on our previous work (Henke et al., 2018; Purwanto et al., 2018), a modular approach was followed: (1) supply of isoprenoid precursor FPP and its conversion to DPP, (2) supply of aromatic precursor pHBA, and (3) prenylation of pHBA followed by sequential modification of the aromatic moiety leading to CoQ10. Establishing CoQ10 production in a microorganism naturally lacking ubiquinone was achieved.

## Materials and Methods

### Bacterial Strains and Growth Conditions

Bacterial strains used in this study are listed in Table 1. *E. coli* DH5α (Hanahan, 1983) was used for plasmid construction. *C. glutamicum* ATCC 13032 was used as platform strain for metabolic engineering. *E. coli ubi* mutants were used for experimental verification of plasmid expression. Pre-cultures of *E. coli* and *C. glutamicum* were performed in lysogeny broth (LB) and brain heart infusion (BHI) medium at 37°C and 30°C in baffled shake flasks on a rotary shaker at 160 rpm and 120 rpm, respectively. Cultures were inoculated from fresh LB agar plates. For growth and production experiments, *C. glutamicum* cells from pre-cultures were washed once with TN buffer pH 6.3 (10 mM Tris–HCl, 150 mM NaCl), inoculated to an optical density at 600 nm (OD_600_) of 1 in CGXII minimal medium (Eggeling and Bott, 2005) with 40 g L^–1^ glucose as sole carbon source and incubated at 30°C and 120 rpm (shaking diameter 16.5 cm). OD_600_ was measured using a V-1200 spectrophotometer (VWR, Radnor, PA, United States). For amorpha-4,11-diene (amorphadiene) production, 10% (v/v) of dodecane and 1 mM of isopropyl-β-D-1-thiogalactopyranoside (IPTG) were added to the minimal medium after 6 hours of cultivation to capture the volatile product. The *E. coli ubi* mutant complementation experiment was performed in an M9 minimal medium (Sambrook et al., 1989) with 20 mM of succinate as sole carbon source. LB pre-cultures were washed once in TN buffer, inoculated to an OD_600_ of 0.1 or 0.01 in 3 mL of M9 minimal medium in Duetz plates (Duetz et al., 2000) and incubated at 37°C and 220 rpm. When necessary, kanamycin (25 μg mL^–1^), spectinomycin (100 μg mL^–1^), and tetracycline (5 μg mL^–1^) were added to the medium. To induce gene expression from the vectors pVWEx1 (Peters-Wendisch et al., 2001), pEKEx3 (Stansen et al., 2005), and pEC-XT99A (Kirchner and Tauch, 2003), 1 mM of IPTG was added. For expression of *ddsA* and *ubiA* from pRG_Duet2 (Gauttam et al., 2019), 1 mM of IPTG and 0.25 μg mL^–1^ of anhydrotetracycline (ATc) were added, respectively.

**TABLE 1:** Strains used in this work.

Strains	Description	Source
***C. glutamicum***		
WT	Wild type, ATCC 13032	ATCC
WT (pVWEx1)	WT carrying pVWEx1	This work
WT (pVWEx1-*ddsA*)	WT carrying pVWEx1-*ddsA*	This work
WT (pVWEx1-ADS)	WT carrying pVWEx1-ADS	This work
Δ*crtOP*Δ*idsA*Δ*crtB2I’I2*	WT carrying deletion of *crtOP* (cg0717-cg0723), *idsA* (cg2384), and *crtB2I’I2* (cg2668-cg2672)	Henke et al., 2018
UBI000	LP4::*P_*tuf*_-ispA* mutant of *C. glutamicum* Δ*crtOP*Δ*idsA*Δ*crtB2I’I2*	This work
UBI000 (pVWEx1-ADS)	UBI000 carrying pVWEx1-ADS	This work
UBI000 (pEC-XT99A)	UBI000 carrying pEC-XT99A	This work
UBI000 (pEC-XT99A-*ubiDIBX*)	UBI000 carrying pEC-XT99A-*ubiDIBX*	This work
UBI000 (pEKEx3)	UBI000 carrying pEKEx3	This work
UBI000 (pEKEx3-*ubiGHEF*)	UBI000 carrying pEKEx3-*ubiGHEF*	This work
UBI100	Δ*pobA* mutant of UBI000	This work
UBI200	Δ*pcaHG*::*P_*sod*_-ubiC^*FBR*^* mutant of UBI100	This work
UBI300	Δ*vdh*::*P_*ilvC*_-aroG^*FBR*^* mutant of UBI200	This work
UBI400	Δ*qsuABCD*::*P_*tuf*_-qsuC* mutant of UBI300	This work
UBI401	UBI400 carrying pVWEx4	This work
UBI405	UBI400 carrying pRG_Duet2-*ddsA-ubiA*	This work
UBI412	UBI400 carrying pRG_Duet2-*ddsA-ubiA* and pEC-XT99A-*ubiDIBX*	This work
UBI413	UBI400 carrying pRG_Duet2-*ddsA-ubiA*, pEC-XT99A-*ubiDIBX* and pEKEx3-*ubiGHEF*	This work
***E. coli***		
K-12	K-12 MG1655 wild type, ATCC 47076	ATCC
DH5α	*F-thi-1 endA1 hsdr17(r-, m-) supE44 1lacU169 (Φ80lacZ1M15) recA1 gyrA96*	Hanahan, 1983
S17-1	*recA pro hsdR* RP4-2-Tc::Mu-Km::Tn7	Simon et al., 1983
Δ*ubiG*	F-, Δ*(araD-araB)567*, Δ*lacZ4787*(::rrnB-3), λ^–^, Δ*ubiG785::kan, rph-1*, Δ*(rhaD-rhaB)568, hsdR514*	Baba et al., 2006
Δ*ubiG* (pEKEx3)	Δ*ubiG* carrying pEKEx3	This work
Δ*ubiG* (pEKEx3-*ubiG*)	Δ*ubiG* carrying pEKEx3-*ubiG*	This work
Δ*ubiH*	F-, Δ*(araD-araB)567*, Δ*lacZ4787*(::rrnB-3), λ^–^, Δ*ubiH758::kan, rph-1*, Δ*(rhaD-rhaB)568, hsdR514*	Baba et al., 2006
Δ*ubiH* (pEKEx3)	Δ*ubiH* carrying pEKEx3	This work
Δ*ubiH* (pEKEx3-*ubiG*)	Δ*ubiH* carrying pEKEx3-*ubiG*	This work
Δ*ubiH* (pEKEx3-*ubiGH*)	Δ*ubiH* carrying pEKEx3-*ubiGH*	This work
Δ*ubiE*	F-, Δ*(araD-araB)567*, Δ*lacZ4787*(::rrnB-3), λ^–^, Δ*ubiE778::kan, rph-1*, Δ*(rhaD-rhaB)568, hsdR514*	Baba et al., 2006
Δ*ubiE* (pEKEx3)	Δ*ubiE* carrying pEKEx3	This work
Δ*ubiE* (pEKEx3-*ubiGH*)	Δ*ubiE* carrying pEKEx3-*ubiGH*	This work
Δ*ubiE* (pEKEx3-*ubiGHEF*)	Δ*ubiE* carrying pEKEx3-*ubiGHEF*	This work
Δ*ubiF*	F-, Δ*(araD-araB)567*, Δ*lacZ4787*(::rrnB-3), λ^–^, Δ*ubiF722::kan, rph-1*, Δ*(rhaD-rhaB)568, hsdR514*	Baba et al., 2006
Δ*ubiF* (pEKEx3)	Δ*ubiF* carrying pEKEx3	This work
Δ*ubiF* (pEKEx3-*ubiGH*)	Δ*ubiF* carrying pEKEx3-*ubiGH*	This work
Δ*ubiF* (pEKEx3-*ubiGHEF*)	Δ*ubiF* carrying pEKEx3-*ubiGHEF*	This work

### Recombinant DNA Work and Strain Construction

Standard molecular genetic techniques were performed as described (Green and Sambrook, 2012). Competent *E. coli* DH5α cells were prepared according to the RbCl method and transformation was performed by heat shock (Green and Sambrook, 2012). *C. glutamicum* was transformed via electroporation (Eggeling and Bott, 2005) at 2.5 kV, 200 Ω, and 25 μF. PCR amplification was performed with Phusion High-Fidelity DNA polymerase and ALLin^TM^ HiFi DNA Polymerase according to the manufacturer (New England Biolabs, United Kingdom, or highQu GmbH, Germany) using the primers specified in Table 3. As template for all *ubi* genes, genomic DNA from *E. coli* K-12 MG1655 was used, *ddsA* was amplified from *P. denitrificans* genomic DNA, and the construct *P_*tuf*_-ispA* was amplified from pSH1-*ispA*. For the restriction of plasmids pVWEx1, pEKEx3, pEC-XT99A, and pK19*mobsacB* (Schäfer et al., 1994), *Bam*HI was used. Restriction of the dual-inducible plasmid pRG_Duet2 was carried out with *Bam*HI for insertion of *ddsA* and with *Nhe*I for insertion of *ubiA*. Plasmid construction was performed via Gibson Assembly (Gibson et al., 2009), and plasmids are listed in Table 2. Correctness of constructs was verified by insert sequencing. Deletion and replacement of chromosomal regions were carried out by using the suicide vector pK19*mobsacB* and two-step homologous recombination as described (Heider et al., 2014b). Transfer of the suicide vectors was done by trans-conjugation using *E. coli* S17-1 as donor strain (Eggeling and Bott, 2005), and selection of recombinants was made by kanamycin resistance after the first recombination and sucrose sensitivity after the second recombination. Mutants were verified by PCR and sequencing using the primers specified in Table 3.

**TABLE 2:** Plasmids used in this work.

Plasmids	Description	Source
pVWEx1	Kan^*R*^, *P_*tac*_, lacI^*q*^*, pHM1519 oriV_*Cg*_, *C. glutamicum/E. coli* expression shuttle vector	Peters-Wendisch et al., 2001
pVWEx1-*ddsA*	Kan^*R*^, pVWEx1 overexpressing *ddsA* from *P. denitrificans*	This work
pVWEx1-ADS	Kan^*R*^, pVWEx1 overexpressing ADS from *A. annua*	This work
pSH1-*ispA*	Kan^*R*^, *P_*tuf*_.* pHM1519 oriV_*Cg*_, *C. glutamicum/E. coli* expression shuttle vector overexpressing *ispA* from *E. coli*	This work
pRG_Duet2	Kan^*R*^, *P*_*tac*_, *lacI*^*q*^, *P*_*tetR/tetA*_, *tetR*, pBL1 oriV_*Cg*_, dual-inducible *C. glutamicum/E. coli* expression shuttle vector	Gauttam et al., 2019
pRG_Duet2-*ddsA-ubiA*	Kan^*R*^, pRG_Duet2 overexpressing *ddsA* from *P. denitrificans* (induced by IPTG) and *ubiA* from *E. coli* (induced by ATc)	This work
pEKEx3	Spec^*R*^, *P*_*tac*_, *lacI*^*q*^, pBL1 oriV_*Cg*_, *C. glutamicum/E. coli* expression shuttle vector	Stansen et al., 2005
pEKEx3-*ubiG*	Spec^*R*^, pEKEx3 overexpressing *ubiG* from *E. coli*	This work
pEKEx3-*ubiGH*	Spec^*R*^, pEKEx3 overexpressing *ubiG* and *ubiH* from *E. coli*	This work
pEKEx3-*ubiGHEF*	Spec^*R*^, pEKEx3 overexpressing *ubiG, ubiH, ubiE* and *ubiF* from *E. coli*	This work
pEC-XT99A	Tet^*R*^, *P*_*trc*_, *lacI*^*q*^, pGA1 oriV_*Cg*_, *C. glutamicum/E. coli* expression shuttle vector	Kirchner and Tauch, 2003
pEC-XT99A-*ubiDIBX*	Tet^*R*^, pEC-XT99A overexpressing *ubiD, ubiI, ubiB* and *ubiX* from *E. coli*	This work
pK19*mobsacB*	Km^*R*^, pK19 oriV_*Ec*_, *sacB, lacZα, E. coli/C. glutamicum* shuttle vector for construction of insertion and deletion mutants in *C. glutamicum*	Schäfer et al., 1994
pK19*mobsacB*-LP4::*P_*tuf*_-ispA*	pK19*mobsacB* with a construct for insertion of *ispA* from *E. coli* K-12 under control of *C. glutamicum* promoter *P*_*tuf*_ into CgLP4	This work
pK19*mobsacB*-Δ*pobA*	pK19*mobsacB* with a construct for deletion of *pobA* (cg1226), which has been amplified from APS529 Purwanto et al., 2018 with the primers pobA-fw and pobA-rv	This work
pK19*mobsacB*-Δ*pcaHG*::*P_*sod*_-ubiC^*FBR*^*	pK19*mobsacB* with a construct for deletion of *pcaHG* (cg2631-cg2630) and insertion of *ubiC*^*L31A*^ from *E. coli* K-12 under control of *C. glutamicum* promoter *P*_*sod*_	Purwanto et al., 2018
pK19*mobsacB*-Δ*vdh*::*P_*ilvC*_-aroG^*FBR*^*	pK19*mobsacB* with a construct for deletion of *vdh* (cg2953) and insertion of *aroG*^*D146N*^ from *E. coli* K-12 under control of *C. glutamicum* promoter *P*_*ilvC*_, which has been amplified from APS529 Purwanto et al., 2018 with the primers vdh-fw and vdh-rv	This work
pK19*mobsacB*-Δ*qsuABCD*::*P_*tuf*_-qsuC*	pK19*mobsacB* with a construct for deletion of *qsuABCD* (cg0501-cg0504) and insertion of *qsuC* (cg0503) under control of *C. glutamicum* promoter *P*_*tuf*_	Walter et al., 2020

**TABLE 3:** Primers used in this work.

Primers	Sequence (5′ to 3′)
pVW-ddsA-fw / pRG-ddsA-fw	CCTGCAGGTCGACTCTAGAG**GAAAGGAGGCCCTTCAG**ATGGGCATGAACGAAAACGT
pVW-ddsA-rv / pRG-ddsA-rv	GAGCTCGGTACCCGGGGATCTCAGGACAGGCGCGAGACGA
pRG-ubiA-fw	AGAGGAGGAAAGGGTATACG**GAAAGGAGGCCCTTCAG**ATGGAGTGGAGTCTGACGCA
pRG-ubiA-rv	CAATTTAAATCCTAGGGCTATCAGAAATGCCAGTAACTCA
pG-ubiG-fw	CCTGCAGGTCGACTCTAGAGGTATAC**GAAAGGAGGCCCTTCAG**ATGAATGCCGAAAAATCGC
pG-ubiG-rv	GAGCTCGGTACCCGGGGATCTCACTTATTCTGCGTGTGC
pGH-ubiG-rv	GTAAGTCACTTATTCTGCGTGTGC
pGH-ubiH-fw	GCACACGCAGAATAAGTGACTTACCCGCGG**GAAAGGAGGCCCTTCAG**ATGAGCGTAATCATCGTCGGTGG
pGH-ubiH-rv	GAGCTCGGTACCCGGGGATCTCAACGCGCCACCCAACC
pGHEF-ubiH-rv	AAGAGTCAACGCGCCACCCAACC
pGHEF-ubiE-fw	GGTTGGGTGGCGCGTTGACTCTTCCATGG**GAAAGGAGGCCCTTCAG**ATGGTGGATAAGTCACAAG
pGHEF-ubiE-rv	GTGACTCAGAACTTATAACCACGATGC
pGHEF-ubiF-fw	GCATCGTGGTTATAAGTTCTGAGTCACGCTAGC**GAAAGGAGGCCCTTCAG**ATGACAAATCAACCAACGGAAATTGC
pGHEF-ubiF-rv	GAGCTCGGTACCCGGGGATCCTACAACCCTAACGCATATTTCAG
pDIBX-ubiD-fw	ACACAGGAAACAGACCATGGGCGGCCGC**GAAAGGAGGCCCTTCAG**ATGGACGCCATGAAATATAAC
pDIBX-ubiD-rv	GTAAGTCAGGCGCTTTTACCGTTG
pDIBX-ubiI-fw	CAACGGTAAAAGCGCCTGACTTACACTAGT**GAAAGGAGGCCCTTCAG**ATGCAAAGTGTTGATGTAGC
pDIBX-ubiI-rv	AAGAGTTAACGCAGCCATTCAGG
pDIBX-ubiB-fw	CCTGAATGGCTGCGTTAACTCTTGTTTAAAC**GAAAGGAGGCCCTTCAG**ATGACGCCAGGTGAAGTACG
pDIBX-ubiB-rv	GTGACTCAGCGTGTTTTGCGCCAAC
pDIBX-ubiX-fw	GTTGGCGCAAAACACGCTGAGTCACGCGATCGC**GAAAGGAGGCCCTTCAG**ATGAAACGACTCATTGTAGGCATCAG
pDIBX-ubiX-rv	CCGGGTACCGAGCTCGAATTTTATGCGCCCTGCCAGC
LP4-US-fw	CCTGCAGGTCGACTCTAGAGCCGTTCGGCTGACTCCTTC
LP4-US-rv	CATTCGCAGGGTAACGGCCACATCAAAAAATCCGCCGTTCCTTG
Ptuf-ispA-fw	CAAGGAACGGCGGATTTTTTGATGTGGCCGTTACCCTGCGAATG
ispA-Term-rv	CTTCCGCATCCAAACTCACTTAGTCAAAAGAGTTTGTAGAAACGCAAAAAGG
LP4-DS-fw	CCTTTTTGCGTTTCTACAAACTCTTTTGACTAAGTGAGTTTGGATGCGGAAG
LP4-DS-rv	GAGCTCGGTACCCGGGGATCCTCACTAGTACGCGGATAAATG
LP4-conf-fw	TCCGCTGATTGCAGATGGTC
LP4-conf-rv	GCTCCGACACAGAGTCAATG
pobA-fw	CCTGCAGGTCGACTCTAGAGAAATGCGGTGGTCCAGGCGTAGC
pobA-rv	GAGCTCGGTACCCGGGGATCCCAACCAAAGCCGTCGATAAGGAA
pobA-conf-fw	AAGGCCTGGTGTGAGTGCGTGAAA
pobA-conf-rv	GTACGGCACCTGCGATGAAC
pca-conf-fw	GGCGTACGTACATCAGTGGA
pca-conf-rv	CCCACTGCGGATCAAAAAGG
vdh-fw	CCTGCAGGTCGACTCTAGAGTCGAGGAACACCACGTTGTGG
vdh-rv	GAGCTCGGTACCCGGGGATCATTATTTGGCTGCTCTTCCTCAG
vdh-conf-fw	GCACTTCCCGGAGGCTACCA
vdh-conf-rv	TGCATCTGCTGCAACGGTGG
qsu-conf-fw	GTTCGTGGACAAGTGTGGTGG
qsu-conf-rv	CTACCGCGCGGATTAAACC

*Ribosomal binding sites are in bold, and binding regions of Gibson primers are underlined.*

### Quantification of Amorphadiene

For analysis of amorphadiene production, dodecane supernatants from shake-flask cultivation were analyzed via gas chromatography-mass spectroscopy (GC-MS) as described (Henke et al., 2018) using a TraceGC gas chromatograph (Thermo Scientific, Waltham, MA, United States) and ISQ ion trap mass spectrometer (Thermo Scientific, Waltham, MA, United States) equipped with an AS 3000 autosampler (Thermo Scientific, Schwerte, Germany). A 30 m × 0.25 mm VF-5 column coated with 0.25 μm of 5% diphenyl and 95% of dimethylsiloxane (Varian GmbH, Darmstadt, Germany) was used. Temperatures for injector, interface, and ion source were set to 250°C, 250°C, and 220°C, respectively. 1 μl of sample was injected in splitless mode. Helium was used as carrier gas at 1 mL min^–1^. The oven temperature was set to 80°C for one minute, raised to 120°C at 10°C min^–1^, raised to 160°C at 3°C min^–1^ and further to 270°C at 10 °C min^–1^, held for 2 min. Mass spectra were recorded after the dodecane peak eluted at 12 min with a scanning range of *m/z* 50–750 at 20 scans s^–1^. Chromatograms were evaluated with Xcalibur software version 2.0.7 (Thermo Scientific, Germany). The NIST 05 library (National Institute of Standards and Technology, Gaithersburg, MD, United States; Thermo Finnigan) was used to identify valencene and amorphadiene. Due to the lack of a commercial amorphadiene standard, valencene was used as a standard equivalent and internal standard.

### Crude Extract Preparation and SDS–PAGE

Cultures for crude extract preparation were inoculated in the BHI medium with 1 mM IPTG and antibiotics as described above and grown overnight. Cells were harvested, washed in TN buffer, and stored at −20°C until further use. All following steps were performed on ice. Cells were resuspended in 2 mL TN buffer and sonicated (UP200S, Hielscher Ultrasonics GmbH, Teltow, Germany) for 9 min at an amplitude of 60% and a cycle of 0.5. The lysed cells were centrifuged at 20,200 *g* and 4°C for 60 min. The protein concentration of the supernatant was determined by the Bradford method (Bradford, 1976) with bovine serum albumin as reference. Sodium dodecyl sulfate polyacrylamide electrophoresis (SDS–PAGE) was performed as described (Green and Sambrook, 2012), loading 10 μg of protein samples.

### LC-MS/MS Analysis of Ubi Proteins

To verify the expression of *ubi* genes, proteins were, on the one hand, isolated from induced cells and, on the other hand, excised from SDS–PAGE at their expected positions for LC-MS/MS analysis. Proteins from SDS–PAGE were transferred to tubes which had previously been washed with trifluoroacetic acid:acetonitrile:H_2_O (0.1:60:40 *v*/v) and were digested with trypsin (Trypsin Gold, Promega) overnight as previously described (Shevchenko et al., 2006). For protein isolates, a simple “single-tube” preparation protocol was used (Wang et al., 2005). Bacterial cell pellets were resuspended in 200 μl 100 mM ammonium bicarbonate and subsequently transferred into 2-ml screw caps with 0.5 g of zirconia/silica micro beads of the size 0.01 mm (Bio Spec Products Inc., Bartlesville, OK, United States). Cells were disrupted five times in a Precellys homogenizer (VWR, Darmstadt, Germany) at 6.5 m/s for 30 s. 100 μl of lysed cell extract was mixed with 100 μl of the organic solvent 2,2,2-trifluoroethanol and 5 μl 200 mM of the reducing agent dithiothreitol (DTT) and incubated for 60 min at 60°C. Alkylation of cysteines was performed by adding 20 μl of 200 mM 2-iodoacetamide and incubation for 90 min in the dark at room temperature. Alkylation was stopped by adding 5 ml of 200 mM DTT and incubation for 60 min at room temperature. For tryptic digestion, samples were diluted 1:10 with 50 mM ammonium bicarbonate. Tryptic digestion was performed at 37°C overnight. Digested peptides were purified using Sep-Pak^®^ Vac 1cc C18 columns (Waters, Milford, CT, United States). Peptide quantification was done using NanoDrop^TM^ 2000 (Peqlab).

Peptides were analyzed by a nanoLC (Ultimate 3000, Thermo Fisher Scientific, Germany) coupled to an ESI-Orbitrap MS/MS (QExactive Plus, Thermo Fisher Scientific, Germany). The effective gradient length of the 25-cm Acclaim^TM^ PepMap^TM^ 100 C18 analytical column was adjusted to 60 min from 4 to 30% of 80% acetonitrile and 0.1% formic acid followed by 7 min from 30 to 50% of 80% acetonitrile and 0.1% formic acid at a flow rate of 300 nl min^–1^. All samples were measured in full MS mode using a resolution of 70.000 (AGC target of 3e6 and 64 ms maximum IT). For the dd-MS2, a resolution of 17.500 (AGC target of 2e5 and 200 ms maximum IT) was used.

Data analysis was done using the Proteome Discoverer^TM^ Software version 2.4 (Thermo Fisher Scientific, Germany). A *C. glutamicum* protein database, supplemented with the FASTA sequences of the overexpressed *E. coli* proteins, was used. For protein identification, a digestion enzyme was set to trypsin and the maximum number of missed cleavages was set to two. Carbamidomethylation of cysteine was set as a fixed modification. Variable modifications were set as follows: oxidation of methionine, N-terminal acetylation, and N-terminal loss of methionine. A false discovery rate (FDR) of 0.01 was selected for protein and peptide identification.

### Quantification of Metabolites via HPLC

High-performance liquid chromatography (HPLC) was applied for analysis of total carotenoids, pHBA, and protocatechuate (PCA) and quinones, following different sample preparation and HPLC protocols. For all analytes, the Agilent 1200 series system (Agilent Technologies Deutschland GmbH, Böblingen, Germany) with a precolumn (LiChrospher 100 RP18 EC-5 μ (40 × 4 mm), CS Chromatographie Service GmbH, Langerwehe, Germany), and a main column (LiChrospher 100 RP18 EC-5 μ (125 × 4 mm), CS Chromatographie Service GmbH) was used.

For carotenoid analysis from *C. glutamicum*, 1 mL of the culture was harvested by centrifugation (20,200 *g*, 10 min) and the pellet was treated with 800 μl methanol:acetone (7:3) containing 0.05% butylhydroxytoluol at 60°C for 15 min under continuous shaking. Cell debris was spun down (20,000 g, 10 min), and the supernatant was used for HPLC analysis. The detection was carried out with a diode array detector (DAD) at 470 nm. Methanol/water (9:1) (A) and methanol (B) were used as mobile phase with the following gradient at a flow rate of 1.5 mL min^–1^: 0 min B 100%, 10 min B 0%, 32.5 min B 0%. Total carotenoids were quantified by integration of all detected peaks and calculation of their sum.

For analysis of pHBA and PCA, the supernatant from *C. glutamicum* cultures was collected by centrifugation (20,200 *g*, 10 min) and used for HPLC analysis. The detection was done with a DAD at 254 nm. The mobile phase consisted of 0.1 M sodium acetate pH 3.3 (with 0.03% sodium azide) (A) and methanol (B) with the following gradient: 0 min B 8% at 0.7 mL min^–1^, 10 min B 25% at 1.2 mL min^–1^, 15 min B 25% at 1.2 mL min^–1^.

Extraction of quinones was carried out as described (Tian et al., 2010a). Cells from 2 mL of culture were harvested by centrifugation (20,200 *g*, 10 min), and the pellet was treated with 3 M hydrochloric acid [10.8 mL g^–1^ dry cell weight (DCW)] at 84 °C for 35 min. After centrifugation (10,000 *g*, 1 min) the acid was removed and cell debris was resuspended in 400 μl of water. For extraction, the same amount of petroleum ether was added and mixed vigorously. After centrifugation, the organic phase was collected, followed by a second round of extraction. The solvent was evaporated in a Concentrator plus (Eppendorf AG, Hamburg, Germany), and the residue was dissolved in ethanol for HPLC analysis. Detection was done with a DAD at 275 nm, the UV/visible (Vis) spectrum was recorded in the range from 230 to 450 nm. The mobile phase consisted of ethanol (A) and methanol (B) with the following gradient at a flow rate of 1 mL min^–1^: 0 min B 100%, 10 min B 0%, 20 min B 0%.

### Identification of CoQ10 by HPLC-ESI-MS and NanoESI-Q-TOF-MS

For identification of CoQ10, produced in shake flasks, quinone extracts were prepared as described above and analyzed via LC-ESI-MS. A Thermo Scientific UltiMate 3000 (Thermo Scientific, Germering, Germany) UHPLC system coupled to a microTOF-Q hybrid quadrupole/time-of-flight mass spectrometer (Bruker Daltonics, Bremen, Germany) equipped with an electrospray ionization (ESI) source was used. For separation, 5 μl of the samples and of the CoQ10 standard was injected and separated by an Accucore Polar Premium column (100 × 4.6 mm, 2.6 μm particle size) at 40°C. The mobile phase consisted of ethanol (A) and methanol (B), both with 10 mM of ammonium acetate for the formation of adducts [M + NH4]^+^, with the following gradient at a flow rate of 0.3 mL min^–1^: 0 min B 100%, 10 min B 0%, 30 min B 0%, 35 min B 100%, 50 min B 100%. UV detection was at 280 nm, and the ESI source was operated in positive ionization mode. The temperature of the dry gas and the capillary was set to 180°C. The scan range of the MS was set to *m/z* 300–1500. The software DataAnalysis Version 4.0 SP 5 (Bruker Daltonics, Bremen, Germany) was used.

In addition, extracts were analyzed via static nanoESI-Q-TOF-MS. Prior to that, they were separated by HPLC as described above; fractions were collected and evaporated, then incubated for 30 min with ethanol with 10 mM of ammonium acetate for the formation of adducts and again evaporated. Nano-ESI measurements were performed using a Synapt G2Si Q-IMS-TOF mass spectrometer (Waters GmbH, Manchester, United Kingdom) in resolution mode, interfaced to a nano-ESI ion source. Nitrogen served as both the nebulizer gas and the dry gas for nano-ESI. Nitrogen was generated by a nitrogen generator NGM 11. Samples were dissolved in acetonitrile and introduced by static nano-ESI using *in-house* pulled glass emitters. The mass axis was externally calibrated with fragment ions of GluFib as calibration standard. Scan accumulation and data processing were performed with MassLynx 4.1 (Waters GmbH, Manchester, United Kingdom) on a PC Workstation. Determination of exact masses was performed using centroided data.

### Bioreactor Fermentation of UBI413

Batch fermentation of *C. glutamicum* UBI413 was performed in a volume of 2 L in a bioreactor (3.7 L KLF, Bioengineering AG, 8636 Wald, Switzerland) at 30°C and an aeration rate of 2 NL min^–1^. The relative dissolved oxygen saturation (rDOS) was maintained at 30% by control of stirrer frequency. The pH was maintained at pH 7.0 with 4 M KOH and 10% phosphoric acid (w/w). Antifoam 204 (1.2 mL) was added to the medium initially; during growth, it was added manually when necessary. Samples were taken every 8 h and cooled down to 4°C until further use. For inoculation, the first pre-culture of *C. glutamicum* UBI413 was grown in the LB medium; cells were transferred to a second pre-culture in CGXII pH 7.0 with 40 g L^–1^ glucose with the required antibiotics and without IPTG and ATc. The culture was spun down and resuspended in a small volume of fresh CGXII medium to inoculate the bioreactor medium to an OD_600_ of 1.0. The bioreactor medium consisted of CGXII medium without 3-(*N*-morpholino) propanesulfonic acid (MOPS), supplemented with 0.5 g L^–1^ of L-methionine, with antibiotics and 1 mM of IPTG and 0.25 μg mL^–1^ of ATc.

## Results

### Functional Expression of DPP Synthase Gene *ddsA* From *P. denitrificans* in *C. glutamicum*

**FIGURE 2:**
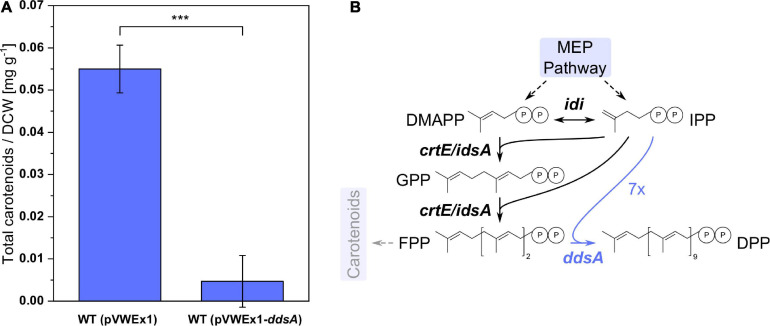
Carotenoid synthesis in *C. glutamicum* WT with and without expression of *ddsA*. **(A)** Total carotenoid amount per dry cell weight from WT (pVWEx1) and WT (pVWEx1-*ddsA*). **(B)** Pathway of isoprenoids with diverging reactions from FPP to DPP and carotenoids. The heterologous expression of *ddsA* is highlighted in blue. MEP, 2-C-methylerythritol 4-phosphate; IPP, isopentenyl diphosphate; DMAPP, dimethylallyl diphosphate; GPP, geranyl diphosphate; FPP, farnesyl diphosphate; DPP, decaprenyl diphosphate; *idi*, isopentenyl diphosphate isomerase; *crtE*, geranylgeranyl diphosphate synthase; *idsA*, geranylgeranyl diphosphate synthase; *ddsA*, decaprenyl diphosphate synthase from *P. denitrificans*. Values and error bars represent means and standard deviations (*n* = 3). Statistical significance is based on a two-sided unpaired Student’s *t*-test (***: *p* < 0.001).

Ubiquinones with different isoprenoid side-chain lengths exist. The chain length of the isoprenoid side chain is determined by biosynthesis of the precursor isoprenoid diphosphate prior to prenylation of pHBA. Therefore, in order to produce CoQ10 with its decaprenyl side chain, DPP synthase DdsA from *P. denitrificans* that converts FPP to DPP was chosen. *C. glutamicum* possesses two isoprenoid synthases, IdsA and CrtE, that convert IPP and DMAPP to GGPP via GPP and FPP (Heider et al., 2014a), and GGPP serves as precursor for the biosynthesis of the carotenoid pigment decaprenoxanthin in *C. glutamicum*. Expression of *ddsA* from *P. denitrificans* was expected to reduce decaprenoxanthin biosynthesis since DdsA competes with carotenogenesis for the isoprenoid diphosphates FPP, DMAPP, and IPP. *C. glutamicum* WT (pVWEx1) and WT (pVWEx1-*ddsA*) were cultivated in CGXII medium for 24 h, followed by carotenoid extraction and HPLC analysis (Figure 2). The expression of *ddsA*, which was verified by SDS-PAGE (data not shown), decreased carotenoid synthesis around 12-fold. Although DPP production could not be directly verified by analytical methods, a decrease of carotenoid synthesis may indirectly confirm functional expression of *ddsA* from *P. denitrificans*.

### Metabolic Engineering for Improved Supply of Isoprenoid Precursor FPP

To avoid competition for isoprenoid diphosphates by carotenogenesis, the strain *C. glutamicum* Δ*crtOP*Δ*idsA*Δ*crtB2I’I2* (Henke et al., 2018) that produces no carotenoids but synthesizes IPP and DMAPP in the MEP pathway was chosen for heterologous expression of FPP synthase gene *ispA* from *E. coli*. The gene *ispA* was expressed from the chromosome from promoter *P*_*tuf*_ by insertion into landing pad 4 of the *C. glutamicum* chromosome (Lange et al., 2018), yielding strain UBI000. Since a direct quantification of FPP was not possible, we chose an indirect method, i.e., by conversion to amorphadiene. This precursor molecule of the antimalarial drug artemisinin is generated from FPP by amorphadiene synthase (ADS) from *Artemisia annua*. Plasmid-borne expression of the ADS gene yielded *C. glutamicum* strains WT (pVWEx1-ADS) and UBI000 (pVWEx1-ADS), respectively.

**FIGURE 3:**
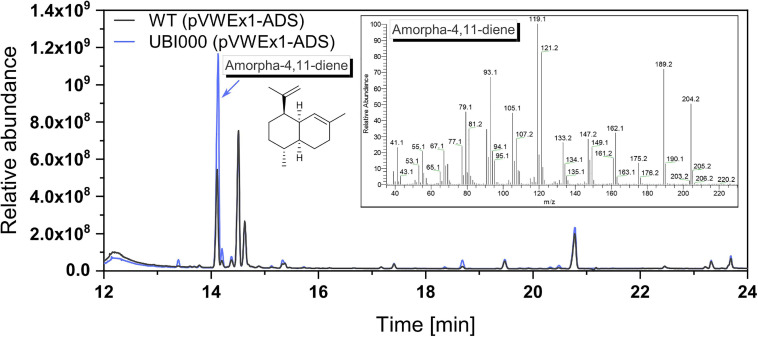
Production of amorphadiene as a means to quantify FPP supply. The extracts of dodecane overlay cultivation with WT (pVWEx1-ADS), shown in black, and UBI000 (pVWEx1-ADS), shown in blue, were analyzed via GC-MS. For quantification, valencene was used as a standard equivalent. Shown are the chromatogram and in the insert the mass spectrum of produced amorphadiene.

These strains were cultivated in the CGXII medium with an overlay of 10% dodecane (v/v) to capture the volatile product amorphadiene. After 75 h of shake-flask cultivation, the dodecane phase was separated from the medium and samples were analyzed by GC-MS analysis (Figure 3). GC-MS confirmed production of amorphadiene. For its quantification, valencene was used as a chemically similar standard equivalent as amorphadiene could not be purchased. It could be shown that UBI000 (pVWEx1-ADS) produced 10.9 ± 0.1 mg L^–1^ valencene equivalents of amorphadiene, thus 2.5 times more than WT (pVWEx1-ADS). This indicated an increased FPP supply in the strain UBI000 (pVWEx1-ADS).

### Metabolic Engineering for Improved Supply of Aromatic Precursor pHBA

The aromatic ring of CoQ10 originates from pHBA, a product of the shikimate pathway synthesized naturally by *E. coli*, but not by *C. glutamicum*. Following our previously developed metabolic engineering strategy to enable pHBA production by *C. glutamicum* (Purwanto et al., 2018), the CoQ10 base strain UBI000 was engineered for increased supply of pHBA. First, *pobA*, encoding pHBA hydroxylase, was deleted to avoid degradation of pHBA to PCA, resulting in the strain UBI100. Second, UBI200 was constructed by integration of feedback-resistant (L31A) chorismate-pyruvate lyase gene *ubiC*^*FBR*^ from *E. coli* under control of the promoter *P*_*sod*_ by simultaneous deletion of *pcaHG*, encoding PCA dioxygenase. It was reported that deletion of *pcaHG* together with deletion of *qsuB* led to a significantly reduced PCA production compared to *qsuB* deletion alone (Kallscheuer et al., 2016). To generate increased flux into the shikimate pathway and reduced by-product formation, UBI300 was engineered by integration of *aroG*^*FBR*^, which codes for feedback-resistant (D146N) 3-deoxy-D-arabinoheptulosonate 7-phosphate (DAHP) synthase from *E. coli*, with the simultaneous deletion of *vdh*, coding for vanillin dehydrogenase. Fourth, the operon *qsuABCD*, encoding putative shikimate importer, 3-dehydroshikimate dehydratase, 3-dehydroquinate dehydratase, and shikimate dehydrogenase, was deleted and *qsuC* was overexpressed from the *P*_*tuf*_ promoter to reduce formation of PCA as by-product, yielding strain UBI400.

**FIGURE 4:**
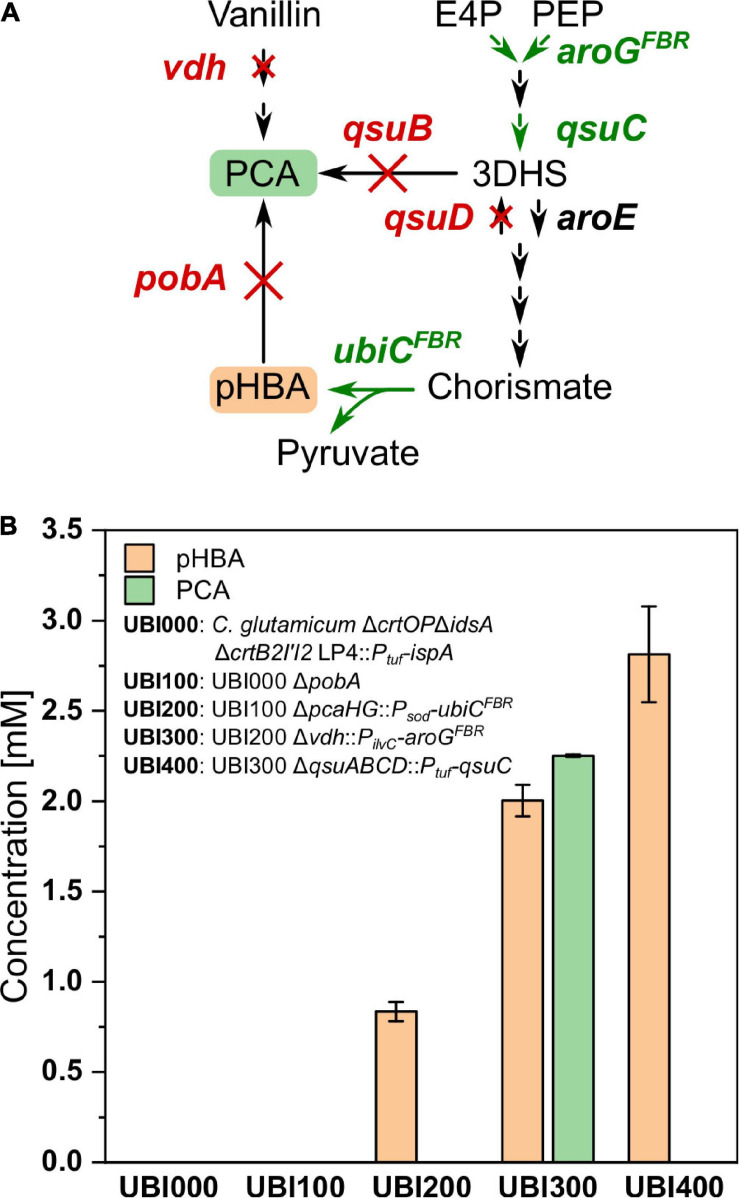
Production of pHBA from consecutively constructed UBI strains. **(A)** Pathway from the intermediates E4P and PEP toward pHBA and PCA with deleted genes shown in red and chromosomal overexpression of genes shown in green. **(B)** Production of pHBA and the side product PCA after 24 h of cultivation. E4P, erythrose 4-phosphate; PEP, phosphoenolpyruvate; 3DHS, 3-dehydroshikimic acid; PCA, protocatechuic acid; pHBA, *para*-hydroxybenzoic acid; *aroG*^*FBR*^, feedback-resistant DAHP synthase from *E. coli*; *qsuC*, 3-dehydroquinate dehydratase; *qsuB*, 3-dehydroshikimate dehydratase; *qsuD*, shikimate dehydrogenase; *aroE*, shikimate dehydrogenase; *ubiC*^*FBR*^, feedback-resistant chorismate-pyruvate lyase from *E. coli*; *pobA, para*-hydroxybenzoate hydroxylase; *vdh*, vanillin dehydrogenase. Values and error bars represent means and standard deviations (*n* = 3).

To compare the production of pHBA and formation of the by-product PCA, the strains UBI000, UBI100, UBI200, UBI300, and UBI400 were cultivated in CGXII medium and supernatants were analyzed after 24 h by HPLC (Figure 4). As expected, strains UBI000 and UBI100 did not produce pHBA because they lack *ubiC*. As a result of the integration of *ubiC*, UBI200 accumulated 0.9 ± 0.1 mM of pHBA and 0.1 ± 0.0 mM of PCA. Production of pHBA by UBI300 doubled due to an increased flux into the shikimate pathway, but accumulation of PCA as by-product exceeded pHBA production. PCA accumulation was almost abolished using strain UBI400 that carried a *qsuB* deletion and *qsuC* overexpression, and pHBA production by this strain was increased to 2.5 ± 0.1 mM (Figure 4). These results suggested that supply of the precursor pHBA in strain UBI400 was sufficient to synthesize CoQ10 to a similar concentration.

### Functional Expression of Polyprenyl Transferase Gene *ubiA* From *E. coli*

For expression of polyprenyl transferase *ubiA* from *E. coli*, this gene and *ddsA* were cloned into the dual-inducible expression vector pRG_Duet2 (Gauttam et al., 2019). This vector allowed for independent expression of both genes: IPTG inducible expression of *ddsA* and ATc inducible expression of *ubiA*.

**FIGURE 5:**
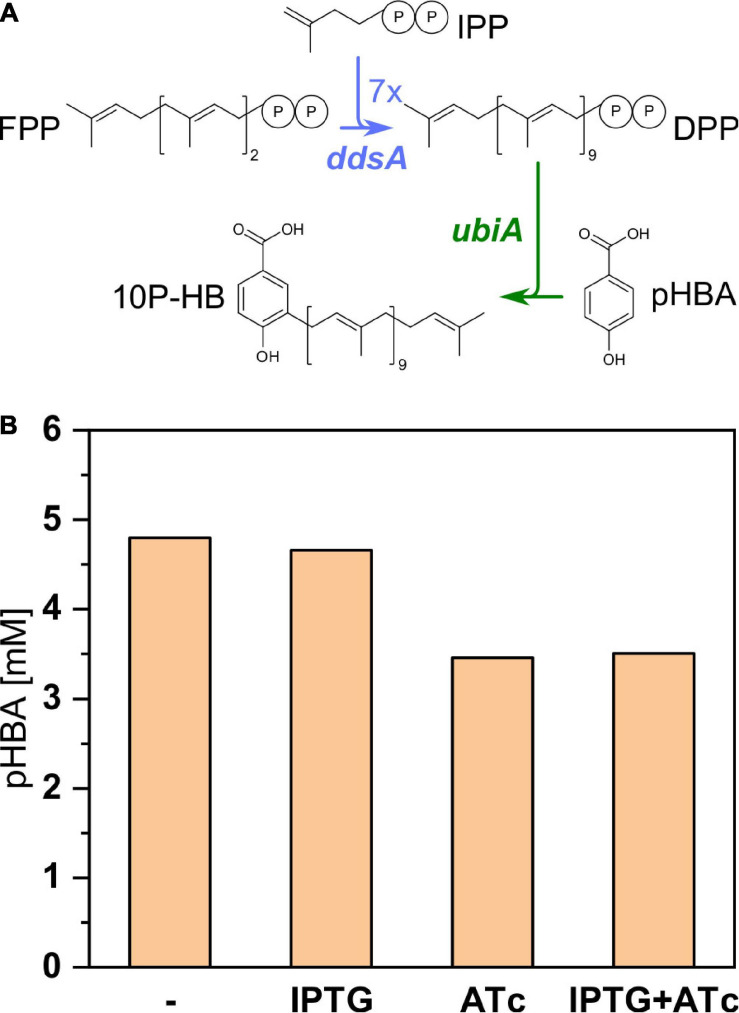
Influence of *ubiA* expression on pHBA production by the strain UBI400 (pRG_Duet2-*ddsA-ubiA*). **(A)** Pathway from the precursor FPP toward the intermediate 10P-HB with heterologously expressed *ddsA* and *ubiA* marked in blue and green, respectively. **(B)** The expression of *ubiA* was induced or not induced via addition of ATc either with or without the additional induction of *ddsA* expression via addition of IPTG. The concentration of pHBA was measured in the respective supernatants. IPP, isopentenyl diphosphate; FPP, farnesyl diphosphate; DPP, decaprenyl diphosphate; DPP, decaprenyl diphosphate; 10P-HB, 3-decaprenyl-4-hydroxybenzoate; *ddsA*, decaprenyl diphosphate synthase from *P. denitrificans*; *ubiA*, 4-hydroxybenzoate octaprenyltransferase from *E. coli*.

Since UbiA condenses pHBA with decaprenyl diphosphate to 3-decaprenyl-4-hydroxybenzoate (10P-HB), pHBA accumulation was examined to estimate if UbiA from *E. coli* was active in recombinant *C. glutamicum.* UBI400 (pRG_Duet2-*ddsA-ubiA*) was cultivated in the CGXII minimal medium in the presence or absence of the inducers IPTG and/or ATc (Figure 5). Without any inducer and with IPTG only, around 4.7 mM pHBA accumulated after 48 h. In the presence of ATc (alone or with IPTG), only 3.5 mM pHBA accumulated in the supernatant. It was expected that pHBA would be prenylated by the activity of UbiA, decreasing the intracellular pHBA concentration and, as consequence, secretion of pHBA to the culture medium. The finding that pHBA accumulation in the culture medium decreased upon *ubiA* expression was considered as indirect evidence for functional expression of polyprenyl transferase gene *ubiA* from *E. coli* in the recombinant *C. glutamicum* strain. IPTG induction of *ddsA* expression did not reduce pHBA further, which may indicate that supply of pHBA is not limiting its prenylation.

### Functional Expression of Genes for the Ubiquinone Modification Pathway

**FIGURE 6:**
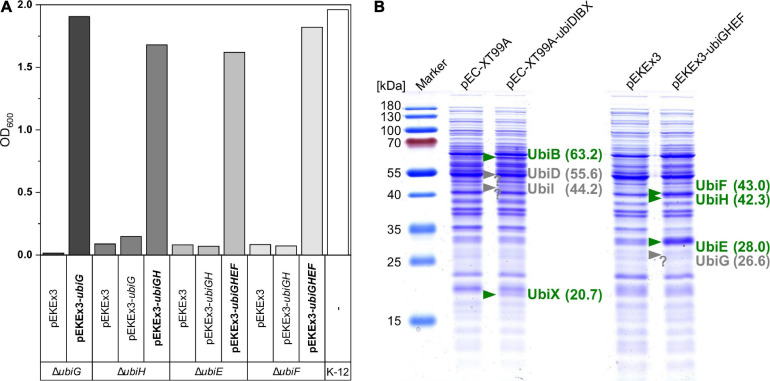
Verification of *ubi* gene function and expression. **(A)** *E. coli* deletion mutants were transformed with control vectors and complementation vectors (bold) and inoculated into M9 minimal medium with 20 mM succinate as sole carbon source. Complementation enabled growth after 45/48 h as can be compared with the positive control strain K-12. **(B)** SDS–PAGE of UBI000 crude extracts carrying different vectors for expression analysis. Confirmed expression based on theoretical protein weights is depicted in green, missing bands are shown in gray at the expected position.

After condensation of pHBA with decaprenyl diphosphate to 10P-HB, CoQ10 biosynthesis proceeds via a number of reactions modifying the aromatic ring. The respective *ubi* genes from *E. coli* were cloned into the *C. glutamicum/E. coli* shuttle vectors pEC-XT99A and pEKEx3. The resulting vectors pEC-XT99A-*ubiDIBX* and pEKEx3-*ubiGHEF* are compatible and allow for IPTG inducible gene expression in *C. glutamicum* as well as *E. coli*. The vectors were tested in genetic complementation experiments since samples of CoQ10 pathway intermediates are scarce, if available at all, and only few enzyme assays have been developed (Poon et al., 1999; Cheng and Li, 2014). Genetically defined ubiquinone biosynthesis mutants of *E. coli* are available, and these mutants are unable to catabolize succinate as sole carbon and energy source (Wallace and Young, 1977). The constructed vectors pEC-XT99A-*ubiDIBX* and pEKEx3-*ubiGHEF* and their derivatives were used to test for genetic complementation of the *ubi* mutants *E. coli ubiD*^*G452R*^, *ubiI*::*kan, ubiX*::*kan, ubiG*::*kan, ubiH*::*kan, ubiE*::*kan*, and *ubiF*::*kan*. These strains and *E. coli* MG1655 as a control strain were grown in M9 medium with succinate as sole carbon source. The mutants *ubiG*::*kan, ubiH*::*kan, ubiE*::*kan*, and *ubiF*::*kan* only grew with succinate as sole carbon source if they carried the respective vector for complementation (Figure 6A). The *E. coli* mutants *ubiI*::*kan* and *ubiX*::*kan* were able to grow without complementation; thus, they were not informative to judge functional expression of *ubiI* and *ubiX* from plasmid pEC-XT99A-*ubiDIBX*. *E. coli ubiD*^*G452R*^ did not grow within 48 h, even when complemented with pEC-XT99A-*ubiD*. We speculate that expression of *ubiD* was too low or that the mutation *ubiD*^*G452R*^ is dominant negative (Figure 6A). Therefore, the identity of Ubi proteins was analyzed by peptide mass fingerprints after excision of protein bands from SDS-PAGE gels, digestion with trypsin, and LC-MS/MS analysis (Supplementary Table 2). While UbiD, UbiI, UbiB, UbiX, UbiG, UbiH, and UbiF could be detected, detection of UbiE was unsuccessful. In addition, proteins were isolated from bacterial cells and whole-proteome LC-MS/MS analysis confirmed presence of all Ubi proteins including UbiE in the respective strains as well as absence of Ubi proteins from the empty vector control strains. In summary, evidence for expression of all cloned *ubi* genes was gained.

### CoQ10 Biosynthesis by Metabolically Engineered *C. glutamicum*

After having established provision of FPP and pHBA as precursors, as well as functional expression of genes for prenylation of pHBA and for modification of the condensation product 10-HB to CoQ10, all engineered pathways were assembled stepwise in a series of recombinant *C. glutamicum* strains: UBI401, UBI405, UBI412, and UBI413 (Table 1). UBI401 is an empty vector control, UBI405 carried only pRG_Duet2-*ddsA-ubiA*, UBI412 possessed pRG_Duet2-*ddsA-ubiA*, and pECXT-*ubiDIBX*, and UBI413 carried three vectors, pRG_Duet2-*ddsA-ubiA*, pECXT-*ubiDIBX*, and pEKEx3-*ubiGHEF*. Strains UBI405 and UBI412 were expected to synthesize 3-decaprenyl-4-hydroxybenzoate and/or 2-decaprenyl-6-hydroxyphenol, whereas strain UBI413 was expected to synthesize CoQ10.

**FIGURE 7:**
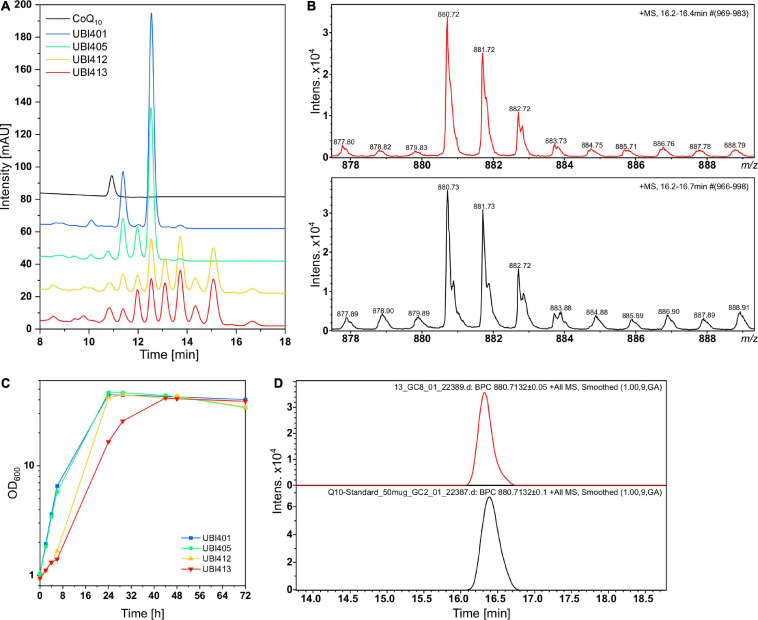
High performance liquid chromatography (HPLC) and LC-MS analyses of quinone extracts from different UBI strains for CoQ10 detection. **(A)** Overlaid chromatograms of the CoQ10 standard and UBI strains with UBI413 (red) carrying the vectors pRG_Duet2-*ddsA-ubiA*, pEC-XT99A-*ubiDIBX*, and pEKEx3-*ubiGHEF* to facilitate CoQ10 production. **(B)** Isotope pattern of CoQ10 from quinone extract of UBI413 (red) and from the CoQ10 standard (black). **(C)** Growth curves of strains that were used for quinone extraction. **(D)** Base peak chromatogram of *m/z* 880.71, the ammonium adduct mass of CoQ10 in the quinone extract of UBI413 (red) and in the CoQ10 standard (black).

*C. glutamicum* strains UBI401, UBI405, UBI412, and UBI413 were cultivated in CGXII medium for 72 h, followed by extraction of quinones from whole cells and HPLC analysis (Figure 7A). While all strains grew to similar biomass concentrations with ODs of 40 to 45, the specific growth rates of strains UBI413 and UBI412 with 0.08 and 0.10 h^−1^ were considerably lower than of UBI401 and UBI405 with 0.30 h^−1^ (Figure 7C). The slowed growth of strains UBI412 and UBI413 may indicate interference of CoQ10 or its precursors with the native respiratory chain. As expected, extracts of the control strain UBI401 carrying an empty vector showed two major peaks in the chromatogram that likely are the native dihydromenaquinones MK9(H2) and MK8(H2). These reduced menaquinones have previously been described to occur in corynebacteria (Collins and Jones, 1981), and they do occur in all tested strains, but at reduced levels in strains UBI405, UBI412, and UBI413. Extracts of strain UBI405 contained two additional compounds, likely MK10(H2) as result of the expression of *ddsA*, and the CoQ10 biosynthesis intermediate 3-decaprenyl-4-hydroxybenzoate, the condensation product of decaprenyl diphosphate, and pHBA generated by UbiA. Extracts of strain UBI412, which further expressed the genes *ubiDIBX*, showed several additional peaks, likely early intermediates of CoQ10 biosynthesis. Alternatively, their prenyl side-chain lengths may be shorter (8 or 9 instead of 10) since pHBA prenyltransferase UbiA is promiscuous for prenyl diphosphates of different lengths and *C. glutamicum* possesses a putative octaprenyl diphosphate synthase encoded by *ispB* (Heider et al., 2014a).

Surprisingly, chromatograms of extracts of strain UBI413 did not contain an extra peak although having been engineered for the complete CoQ10 biosynthesis. One of the peaks present in extracts of strains UBI412 and UBI413 co-eluted with the CoQ10 standard. Their absorption spectra, however, differed from that of CoQ10 (Supplementary Figure 2). The non-identity of the UV spectra of the standard and the CoQ10 formed by strain UBI413 is striking. We hypothesize that at least one other compound co-elutes with CoQ10. Addition of reducing or oxidizing agents to the extracts had no effect on the chromatograms or UV spectra, which supports the notion that the standard and the CoQ10 formed by strain UBI413 do not differ by the oxidation states. Therefore, the samples and the standard were subjected to LC-MS analysis (Figures 7B,D). Extracts of strain UBI413, but not those of the tested other strains, revealed a mass of 880.72 for the peak co-eluting with the CoQ10 standard, which corresponded to CoQ10 [M+NH_4_]^+^. The results were confirmed in another independent shake-flask experiment with subsequent Q-TOF-MS analysis of the quinone extracts. The LC-MS analysis did not provide a clue on the nature of the compound co-eluting with CoQ10, which, thus, remains elusive.

Moreover, to prevent that *S*-adenosyl-L-methionine (SAM)-dependent methylation reactions may limit CoQ10 biosynthesis, 0.5 g L^–1^ of L-methionine was added. Addition of L-methionine has been shown to improve vanillin production in *E. coli* by increased SAM availability (Kunjapur et al., 2016). Indeed, addition of L-methionine altered the ratios of compounds present in quinone extracts including a substance eluting with/near CoQ10, which indicated that flux in the ubiquinone pathway was altered by increased SAM availability (Supplementary Figure 3).

**FIGURE 8:**
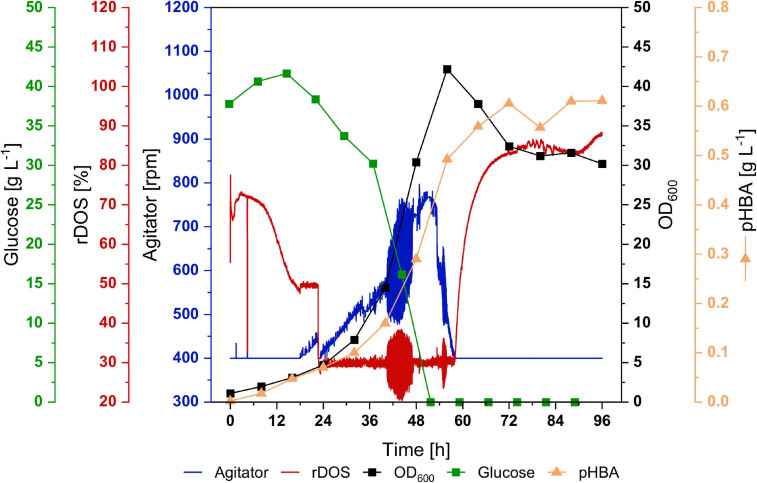
Batch fermentation of *C. glutamicum* UBI413 in 2-L scale. The cultivation was performed in CGXII medium with 40 g L^–1^ glucose, supplemented with 0.5 g L^–1^ of L-methionine, antibiotics, 1 mM of IPTG and 0.25 μg mL^−1^ of ATc for induction of gene expression at 30°C, pH 7.0 and an aeration rate of 2 NL min^−1^. The rDOS was maintained at 30% by control of agitator frequency. Glucose concentration (green) and OD_600_ (black) are shown in squares, rDOS (red) and agitator frequency (blue) are indicated by lines, production of pHBA is shown in triangles. An upper estimate of CoQ10 production is given in the results.

In order to test if CoQ10 production is stable in a bioreactor, a 2-L batch fermentation with strain UBI413 was performed (Figure 8). Moreover, bioreactor fermentation may provide better cultivation conditions than flask cultivation with respect to molecular oxygen, which is required for CoQ10 biosynthesis since UbiI, UbiH, and UbiF are oxygenases. The strain UBI413 was cultivated for 96 h, rDOS was set to 30%, and the agitator frequency was controlled to keep the rDOS constant. The cells reached an OD_600_ of 42; glucose was consumed after around 56 h. A pHBA titer of 0.6 g L^–1^ (4.4 mM) was reached after 72 h, which was comparable with observed titers in shake flasks. Although CoQ10 was not quantifiable due to background peaks, we made an upper estimate: under the assumption that the corresponding peak at the CoQ10 retention time consists only of CoQ10 a titer ≤ 0.43 mg L^–1^ and a cellular content ≤ 36 μg g^–1^ DCW after 96 h were observed.

Taken together, by metabolically engineering non-ubiquinone-containing *C. glutamicum* to produce the precursors DPP and pHBA from extended isoprenoid and shikimate pathways, respectively, and introducing ubiquinone modification genes from *E. coli*, the biosynthesis of CoQ10 was unequivocally documented by the *C. glutamicum* strain UBI413.

## Discussion

Biosynthesis of CoQ10 was established in the non-ubiquinone-containing bacterium *C. glutamicum*. Supply of the aromatic precursor pHBA was enabled following a previously developed strategy (Purwanto et al., 2018). Supply of FPP as isoprenoid precursor upon introduction of the FPP synthase from *E. coli* was demonstrated by production of amorphadiene (10.9 ± 0.1 mg L^–1^ valencene equivalents) when the gene for amorphadiene synthase from *A. annua* was expressed in addition and endogenous carotenogenesis was abolished. Upon expression of heterologous genes for decaprenyl diphosphate synthase from *P. denitrificans* and 4-hydroxybenzoate polyprenyltransferase and ubiquinone modification enzymes from *E. coli*, pHBA was prenylated and CoQ10 was produced as evidenced by MS analysis.

The intracellular concentration of the aromatic precursor pHBA was not measured; however, its supply for CoQ10 biosynthesis appears not to be limiting, since the metabolic engineering strategy followed here to provide pHBA was used previously to establish pHBA overproduction and 19 g L^–1^ (138 mM) pHBA was secreted to the culture medium in a fed-batch fermentation (Purwanto et al., 2018). The finding that strains constructed here secreted up to 5 mM pHBA (Figure 5B) suggested that Co10 biosynthesis was not limited by pHBA supply.

Competition for isoprenoid diphosphates in naturally CoQ10-synthesizing bacteria such as *P. denitrificans* (Yoshida et al., 1998), *Rhodospirillum rubrum* (Tian et al., 2010b), and *R. sphaeroides* was reduced by abolishment/reduction of carotenogenesis, which increased CoQ10 content. However, *R. sphaeroides* suffered from the deletion of the carotenoid gene cluster as the biomass decreased to half of that of the wild type (Zhu et al., 2017). To overcome this detrimental problem (the titer of cell-bound products depends on content per cell as well as on the biomass concentration achieved), it was required to downregulate the carotenoid genes of *R. sphaeroides* by overexpression of the transcriptional repressor gene *ppsR* and of the GGPP synthase gene *crtE* to maintain biomass formation at a higher CoQ10 content (Zhu et al., 2017). By contrast, *C. glutamicum* does not suffer from carotenoid deficiency (data not shown) and therefore constitutes an adequate host for the production of isoprenoid-derived compounds as shown for patchoulol production. The carotenoid-deficient strain *C. glutamicum* Δ*crtOP*Δ*idsA*Δ*crtB2I′I2* (pECXT_*ispA*-*PcPS*)(pVWEx1) produced 60 mg L^–1^ patchoulol and reached a biomass concentration of 4.2 ± 0.6 g L^–1^ (Henke et al., 2018), which is only slightly lower than that of the wild type (5.4 g L^–1^) under the same conditions (Frohwitter et al., 2014), presumably due to the plasmids it carried. Thus, further improving isoprenoid diphosphate precursor supply may be beneficial for CoQ10 production by the strains constructed in this study without compromising biomass formation. In *R. sphaeroides*, this was achieved by self-regulated overexpression of the genes *dxs, dxr, idi*, and *ispD* from the MEP pathway (Lu et al., 2014).

To provide DPP for prenylation of pHBA, we chose to express the heterologous decaprenyl diphosphate synthase gene *ddsA* from *P. denitrificans*. The enzyme has *K*_*m*_ values of 5.0, 0.06, and 2.9 μM for GPP, FPP, and GGPP, respectively, and yields DPP and undecaprenyl diphosphate (UPP), but not shorter diphosphates. The ratio of DPP to UPP synthesized by DdsA depended on the Mg^2+^ concentrations and ranged from 23:1 at 1 mM Mg^2+^ to 5:1 at 5 mM Mg^2+^ (Ishii et al., 1985). In *E. coli*, plasmid-borne expression of *ddsA* alone was sufficient to enable CoQ10 biosynthesis in addition to and independent of native CoQ8 biosynthesis via IspB (Takahashi et al., 2003). In the MK-containing, but CoQ-lacking *C. glutamicum*, we observed a 12-fold reduction of carotenoid content upon expression of *ddsA* (Figure 2), indicating competition for the substrate FPP between DdsA and GGPP synthases IdsA and CrtE on the one hand and competition between DdsA and phytoene synthase CrtB for the substrate GGPP on the other hand. IdsA and CrtE have *K*_*m*_ values of 8 and 0.1 μM for GPP and 20 and 6 for FPP, respectively (Heider et al., 2014a); thus, the fact that DdsA shows higher affinity for both substrates may explain the reduced carotenoid accumulation as consequence of *ddsA* expression. To abolish any competition, the carotenoid-deficient mutant *C. glutamicum* Δ*crtOP*Δ*idsA*Δ*crtB2I’I2* was chosen and the FPP synthase gene *ispA* from *E. coli* was integrated into the chromosome.

For condensation of DPP and pHBA, we chose prenyltransferase UbiA from *E. coli*. When *ubiA* was induced, the amount of pHBA accumulated outside the cells was reduced (Figure 5), which may serve as indirect evidence for functional expression of this prenyltransferase gene. UbiA is highly specific for pHBA, accepting only very similar compounds like 3-chloro-4-hydroxybenzoic acid and 2,4-dihydroxybenzoic acid to some extent (Bräuer et al., 2004); however, these are not known to occur in *C. glutamicum*. By contrast, UbiA is promiscuous for isoprenoid diphosphates of different chain lengths (Suzuki et al., 1994; Cheng and Li, 2014); thus, the product of prenylation depends on the availability of isoprenoid diphosphate species. Biosynthesis of menaquinones MK8 and MK9 in *C. glutamicum* involves prenylation by MenA using either octa- or nonaprenyl diphosphate that are synthesized by the native octa- or nonaprenyl diphosphate synthases such as IspB (Heider et al., 2014a). Hence, it is conceivable that heterologous UbiA accepts octa- or nonaprenyl diphosphate for prenylation of pHBA, yielding CoQ8 and CoQ9 as byproducts of CoQ10 biosynthesis. HPLC analysis revealed that upon expression of *ddsA* and *ubiA* (strain UBI405), new quinone compounds arise, which are absent from the precursor strain and from *C. glutamicum* wild type (data not shown). These compounds could either be pHBA prenylation products CoQ8 and/or CoQ9 or, alternatively, be MK10 synthesized by MenA using DPP instead of octa- or nonaprenyl diphosphate. Thus, selection and/or engineering of prenyltransferases that show as strict substrate specificity for isoprenoid diphosphate substrate as for the aromatic substrate may improve CoQ10 production by decoupling native MK8 and MK9 biosynthesis from engineered CoQ10 biosynthesis.

The product of prenylation of pHBA with DPP, 10P-HB, is modified further by a sequence of enzymes. This process may be limiting. Several studies have shown that UbiG is a bottleneck in ubiquinone biosynthesis and its overexpression increased CoQ10 titers in *E. coli* (Zhu et al., 1995; Zhang et al., 2007) and *R. spharoides* (Lu et al., 2013). In the strain constructed here, UbiG levels may be low since genetic complementation of an *E. coli* Δ*ubiG* mutant by plasmid-borne expression of *ubiG* was successful, whereas SDS–PAGE did not reveal a protein band of UbiG (Figure 6). One option to improve *ubiG* expression is in the form of a membrane-anchored fusion protein of UbiG and UbiE, which improved CoQ10 productivity by recombinant *R. sphaeroides* (Lu et al., 2015). Moreover, certain steps of 10P-HB modification have not been assigned to a protein unambiguously. For instance, an *E. coli* Δ*ubiB* deletion strain accumulates 3-octaprenylphenol and does not produce CoQ8, but the function of UbiB remains unknown (Hajj Chehade et al., 2013). UbiB was believed to catalyze the first monooxygenase step (Poon et al., 2000), which is actually performed by UbiI, and UbiB may function in regulation of ubiquinone synthesis through its putative kinase activity. In our design we included *ubiB* and CoQ10 biosynthesis was successful, but we did not test if *ubiB* is essential for CoQ10 biosynthesis. The CoQ10-synthesizing strain developed here lacked heterologous expression of *ubiJ* and *ubiK*. In *E. coli*, the membrane-bound UbiJ-UbiK complex is believed to facilitate locating the Ubi enzymes at the membrane, where ubiquinone synthesis takes place (Loiseau et al., 2017). Deletion of *ubiK* decreased the CoQ8 content to 18% compared to the wild type, while *ubiJ* was shown to be essential for ubiquinone synthesis in *E. coli* under aerobic conditions (Aussel et al., 2014a). Heterologous expression of *ubiJ* and *ubiK* may improve CoQ10 biosynthesis by recombinant *C. glutamicum*.

While we have achieved a proof-of-concept for biosynthesis of CoQ10 in the first non-ubiquinone-containing bacterium, the demanding pathway may be optimized with regard to (a) efficient provision of DPP, (b) prenylation of pHBA with DPP as sole prenyl diphosphate, and efficient modification of 10P-HB for overproduction of CoQ10 by recombinant *C. glutamicum*.

## Data Availability Statement

The original contributions presented in the study are included in the article/Supplementary Material, further inquiries can be directed to the corresponding author/s.

## Author Contributions

AB, AM, MP, JS, JR, and TP carried out experimental procedures of the present study. AB, AM, MP, JS, TP, PP-W, J-HL, and VW analyzed data. AB prepared a draft of the manuscript. AB and VW finalized the manuscript. J-HL and VW coordinated the study. All authors read and approved the final version of the manuscript.

## Conflict of Interest

The authors declare that the research was conducted in the absence of any commercial or financial relationships that could be construed as a potential conflict of interest.
