# Supplementary material for: Coenzyme Q10 Biosynthesis Established in the Non-Ubiquinone Containing Corynebacterium glutamicum by Metabolic Engineering
Source: Front Bioeng Biotechnol. 2021 Mar 30;9:650961. doi: 10.3389/fbioe.2021.650961 (PMC8042324; doi:10.3389/fbioe.2021.650961)
Supplement: Supplementary file 1 [file Data_Sheet_1.PDF]

Supplementary Material

1 Supplementary Figures

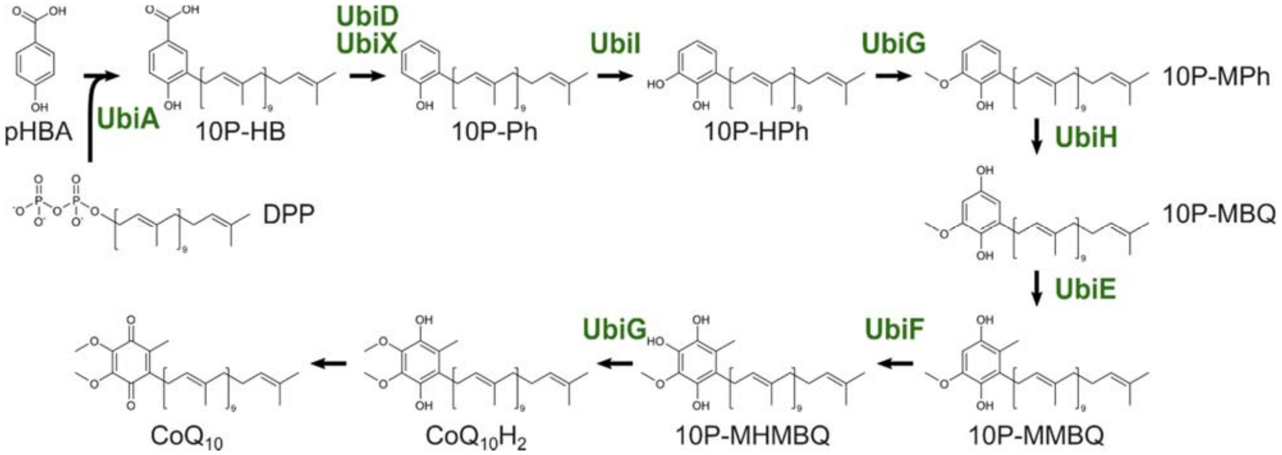

Supplementary Figure S1. Coenzyme Q_{10} biosynthesis pathway with chemical structures. UbiA, 4-hydroxybenzoate octaprenyltransferase; UbiD, 3-octaprenyl-4-hydroxybenzoate decarboxylase; UbiX, flavin prenyltransferase; UbiI, 2-octaprenylphenol hydroxylase; UbiG, 2-octaprenyl-6-hydroxyphenol/2-octaprenyl-3-methyl-5-hydroxy-6-methoxy-1,4-benzoquinol methyltransferase; UbiH, 2-octaprenyl-6-methoxyphenol hydroxylase; UbiE, ubiquinone/menaquinone biosynthesis methyltransferase; UbiF, 2-octaprenyl-3-methyl-6-methoxy-1,4-benzoquinol hydroxylase; UbiB, probably protein kinase, function unknown; pHBA, *para*-hydroxybenzoate; DPP, decaprenyl diphosphate, 10P-HB, 3-decaprenyl-4-hydroxybenzoate; 10P-Ph, 2-decaprenylphenol; 10P-HPh, 2-decaprenyl-6-hydroxyphenol; 10P-MPh, 2-decaprenyl-6-methoxyphenol; 10P-MBQ, 2-decaprenyl-6-methoxy-1,4-benzoquinol; 10P-MMBQ, 2-decaprenyl-3-methyl-6-methoxy-1,4-benzoquinol; 10P-MHMBQ, 2-decaprenyl-3-methyl-5-hydroxy-6-methoxy-1,4-benzoquinol; $CoQ_{10}H_2$, ubiquinol-10; CoQ_{10}, ubiquinone-10.

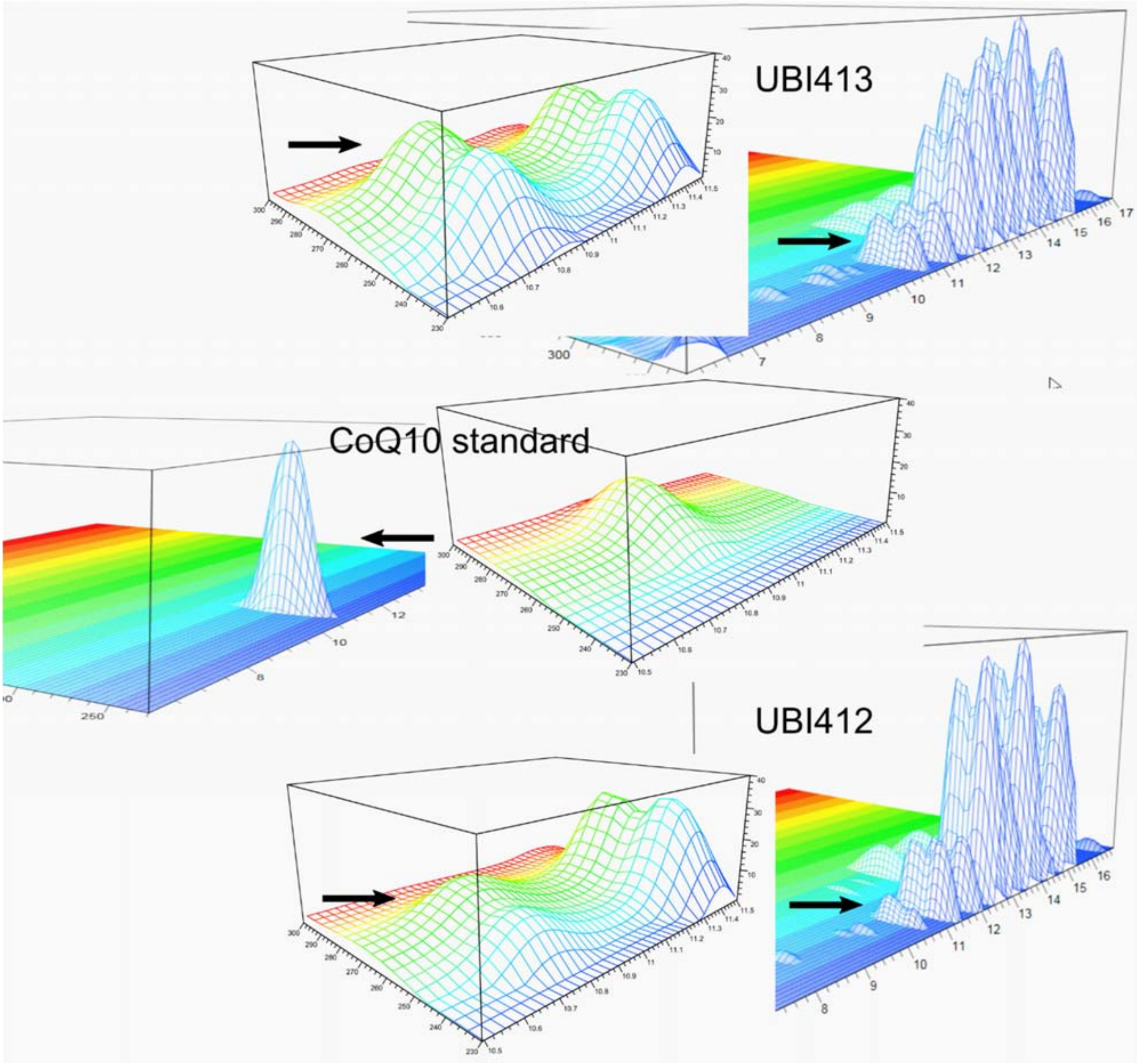

Supplementary Figure S2. 3D-chromatograms from HPLC analysis of a CoQ10 standard and extracts from strains UBI413 and UBI412. The black arrows point at the peaks with the same retention time as CoQ10. Different UV spectra compared to the spectrum of CoQ10 can be observed.

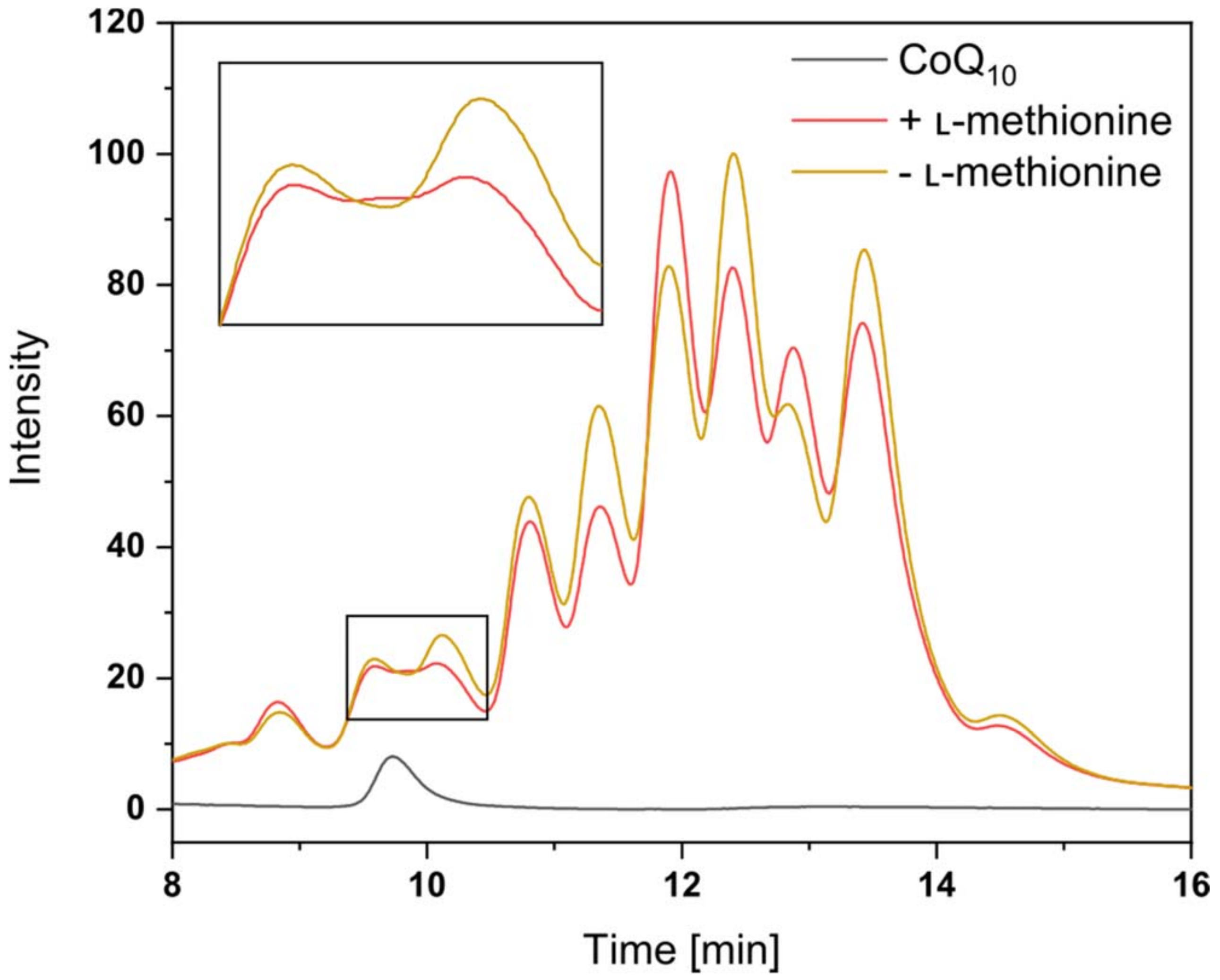

Supplementary Figure S3. Effect of 0.5 g L^{-1} L-methionine addition to CGXII medium on the chromatogram of the quinone extract of strain UBI413. In the inlet the peaks around the retention time of CoQ10 are highlighted.

2 Supplementary Tables

Supplementary Table S1. List of all relevant genes with corresponding enzyme functions.

Gene	Enzyme / Function
crtOP	cg0717-cg0723 (genes for carotenoid biosynthesis)
crtB2I'I2	cg2668-cg2672 (genes for carotenoid biosynthesis)
crtE	geranylgeranyl diphosphate synthase
idsA	geranylgeranyl diphosphate synthase
ispA	farnesyl diphosphate synthase
ddsA	decaprenyl diphosphate synthase from *P. denitrificans*
$aroG^{FBR}$	feedback-resistant DAHP synthase from *E. coli*
qsuB	3-dehydroshikimate dehydratase
qsuC	3-dehydroquinate dehydratase
qsuD	shikimate dehydrogenase
pobA	*para*-hydroxybenzoate hydroxylase
$ubiC^{FBR}$	feedback-resistant chorismate-pyruvate lyase from *E. coli*
ubiA	4-hydroxybenzoate octaprenyltransferase
ubiB	probably protein kinase, function unknown
ubiD	3-octaprenyl-4-hydroxybenzoate decarboxylase
ubiX	flavin prenyltransferase
ubiI	2-octaprenylphenol hydroxylase
ubiG	2-octaprenyl-6-hydroxyphenol/2-octaprenyl-3-methyl-5-hydroxy-6-methoxy-1,4-benzoquinol methyltransferase
ubiH	2-octaprenyl-6-methoxyphenol hydroxylase
ubiE	ubiquinone/menaquinone biosynthesis methyltransferase
ubiF	2-octaprenyl-3-methyl-6-methoxy-1,4-benzoquinol hydroxylase

Supplementary Table S2. **LC-MS/MS analysis of whole proteome isolates of UBI000 (pEC-XT99A-*ubiDIBX*) and UBI000 (pEKEx3-*ubiGHEF*) and of excised SDS-PAGE bands from respective strains (last column).** #Peptides, number of distinct peptide sequences identified per protein; #PSMs, peptide spectrum matches: total number of identified peptide sequences for the protein; score, sum of the scores of the individual peptides.

Protein	Protein name	Accession	Coverage [%]	# Peptides	# PSMs	Score (whole proteome)	Score (SDS-PAGE)
UbiD	3-octaprenyl-4-hydroxybenzoate carboxy-lyase	P0AAB4	57	20	30	99	248
UbiI	2-octaprenylphenol hydroxylase	P25535	72	19	34	146	99
UbiB	Probable protein kinase	P0A6A0	64	30	57	163	59
UbiX	Flavin prenyltransferase	P0AG03	76	9	22	72	25
UbiG	Ubiquinone biosynthesis O-methyltransferase	P17993	86	14	98	340	249
UbiH	2-octaprenyl-6-methoxyphenol hydroxylase	P25534	78	19	41	156	105

UbiE	Ubiquinone/menaquinone biosynthesis C-methyltransferase	P0A887	55	10	13	35	n.d.
UbiF	3-demethoxyubiquinol 3-hydroxylase	P75728	64	17	43	166	275
